# Linear DNA expression cassette encapsulated in lipid nanoparticles confers complete protection against influenza A virus in mice

**DOI:** 10.1128/msphere.00135-26

**Published:** 2026-09-04

**Authors:** Danh C. Lai, The N. Nguyen, Giao P. Trinh, Hiep L. X. Vu

**Affiliations:** 1 Nebraska Center for Virology, University of Nebraska-Lincoln 14719, Lincoln, Nebraska, USA; 2 School of Veterinary Medicine and Biomedical Sciences, University of Nebraska-Lincoln 14719, Lincoln, Nebraska, USA; 3 Department of Animal Science, University of Nebraska-Lincoln 14719, Lincoln, Nebraska, USA; Instituto de Biotecnologia/UNAM, Cuernavaca, Morelos, Mexico

**Keywords:** influenza A virus, DNA vaccine, linear DNA expression cassette, lipid nanoparticles

## Abstract

DNA-based vaccines have been developed and tested against a range of infectious agents, including influenza A viruses (IAV). Bacterial plasmid DNA is widely used as a vector for delivering genes encoding vaccine immunogens. However, the inclusion of antibiotic resistance genes in conventional plasmid constructs raises safety concerns. Linear DNA expression cassettes produced via PCR that lack antibiotic resistance genes, and bacterial sequences can serve as a safer alternative vector. In this study, we employed a mouse model of IAV to evaluate the protective efficacy of a lipid nanoparticle (LNP)-encapsulated linear DNA expression cassette containing the hemagglutinin gene of IAV H1N1 produced by PCR amplification. For comparison, a circular plasmid DNA containing the same gene was also constructed. The LNP-encapsulated DNA cassette (LNP-L) exhibited similar physicochemical characteristics, such as particle size, polydispersity index, zeta potential, and encapsulation efficiency, compared to the LNP-encapsulated circular plasmid DNA (LNP-P). However, LNP-L produced significantly lower transgene expression than LNP-P in cell culture. Mice immunized with LNP-L developed a delayed onset of antibody response compared to those immunized with LNP-P, but the magnitudes of both humoral and cell-mediated immune responses were comparable between the two groups after booster immunization. Moreover, mice immunized with LNP-L exhibited both Th1 and Th2 responses, similar to those immunized with LNP-P. Importantly, mice immunized with either LNP-L or LNP-P were fully protected upon challenge with a lethal dose of IAV H1N1. These findings suggest that linear DNA expression cassettes encapsulated in LNPs represent a promising strategy for vaccine development.

**IMPORTANCE:**

Lipid nanoparticle (LNP)-encapsulated DNA vaccines represent a promising platform for the rapid development of vaccines against infectious pathogens. However, the inclusion of an antibiotic-resistance gene in plasmid DNA vectors used to deliver vaccine immunogens raises potential safety concerns. This study explores the feasibility of using a linear expression cassette produced via PCR amplification as an alternative system for delivering vaccine immunogens. Compared with conventional plasmid DNA, the linear expression cassette contains only essential elements, including a promoter, the gene encoding the vaccine immunogen, and a polyadenylation signal, and can be produced in a cell-free system. Using a mouse model for influenza A virus, we demonstrate that an LNP-encapsulated linear DNA cassette induces humoral and cell-mediated immune responses and protects immunized mice against a lethal influenza virus challenge. Thus, the LNP-encapsulated linear expression cassette may provide a safer approach for rapid responses to infectious diseases in animals.

## INTRODUCTION

In the 1990s, it was discovered that intramuscular injection of mice with naked plasmid DNA resulted in expression of the transgene in muscle tissues for up to 2 months (1). Subsequent studies demonstrated that delivery of DNA-coated gold particles into the skin of mice induced antibody responses (2). These landmark findings paved the way for using DNA plasmids in nonviral gene delivery therapies and vaccine development (3). DNA plasmids are noninfectious, relatively easy to produce in large quantities at low cost, and highly stable, even at ambient temperatures (4). These characteristics make DNA-based vaccines particularly suitable for responding to emerging pathogens. Despite these advantages, two major challenges have limited their adoption.

The first challenge involves the presence of an antibiotic resistance gene. Conventional DNA-based vaccines rely on circular bacterial plasmids to deliver the antigen-encoding gene. These plasmids typically contain a bacterial origin of replication and an antibiotic resistance gene, which serves as a selection marker to ensure plasmid maintenance within the bacterium. The presence of antibiotic resistance genes in the plasmid backbone raises concerns about the potential transfer of antibiotic resistance to both pathogenic and commensal microflora in recipients (4). Multiple strategies have been developed to produce DNA plasmids without antibiotic resistance genes (4–7). Additionally, several approaches have been explored to generate synthetic DNA expression cassettes, which contain only three essential components: an enhancer and promoter, the antigen-encoding gene, and a polyadenylation [poly(A)] signal (8–11). As such, the synthetic cassette does not include antibiotic resistance genes or the origin of replication, thus offering a safer alternative solution.

The second challenge stems from the inefficient cellular uptake of naked DNA. In general, direct injection of naked DNA to humans or animals often results in poor immune responses, largely because the naked DNA molecules do not efficiently enter host cells (12, 13). To overcome this limitation, several delivery strategies have been explored, including *in vivo* electroporation, gene guns, and nanoparticle-delivery systems (3, 13).

Lipid nanoparticles (LNPs) have emerged as highly effective carriers for nucleic acid vaccines, including both mRNA and DNA (14, 15). LNPs protect nucleic acid cargo from degradation and facilitate cellular uptake, thus enhancing expression of vaccine immunogens (16). Some studies have also reported that LNPs possess adjuvant properties (17, 18), thereby enhancing both innate and adaptive immune responses following immunization. We and others have demonstrated that encapsulating circular bacterial DNA plasmids with LNPs significantly enhances vaccine immunogenicity in various animal models (19–26). However, the feasibility of encapsulating linear DNA expression cassettes in LNP has not been explored.

In the present study, we evaluated the feasibility of using LNPs to encapsulate linear DNA amplicon generated by PCR. We chose influenza A virus (IAV) as a model to assess the effectiveness of the LNP-encapsulated linear DNA vaccine. IAV is a member of the *Orthomyxoviridae* family. The virus infects a wide variety of hosts, including humans, pigs, and birds, and cross-species transmission has been frequently observed (reviewed in reference 27). In humans, seasonal influenza affects approximately one billion people annually, with 3–5 million cases classified as severe (World Health Organization, http://www.who.int/mediacentre/factsheets/fs211/en/). Pandemics also occur periodically, with at least three major events recorded in the 20th century. The 1918 H1N1 influenza pandemic is estimated to have caused around 50 million deaths worldwide (28). More recently, the 2009 swine-origin H1N1 pandemic caused an estimated 151,700–575,400 deaths globally in its first year (29).

The viral hemagglutinin (HA) is the dominant surface antigen responsible for viral attachment to the cellular receptor (30, 31). HA is therefore a key target for vaccine development. Immunization of animals with HA alone is sufficient to confer complete protection (32). We have demonstrated that LNP-encapsulated circular plasmid DNA encoding HA from H1N1 and H3N2 influenza viruses elicits strong protective immunity in animal models (19, 20).

In this study, we directly compared the physicochemical properties and immunogenicity of LNP-encapsulated linear DNA cassettes (LNP-L) encoding the HA of H1N1 virus with those of LNP-encapsulated circular plasmid DNA (LNP-P) encoding the same antigen. We show that mice immunized with the LNP-L vaccine developed both humoral and cell-mediated immune responses and were protected against a lethal H1N1 virus challenge.

## MATERIALS AND METHODS

### Cells, viruses, and peptides

Human embryonic kidney cells (HEK-293T, CRL-3216) and Madin-Darby canine kidney (MDCK; CCL-34) cells were obtained from the American Type Culture Collection. The cells were cultured in Dulbecco’s modified Eagle medium (DMEM) supplemented with 10% fetal bovine serum at 37°C with 5% CO_2_. The mouse-adapted influenza virus A/California/07/2009 (H1N1)pdm09, designated CA09, was kindly provided by Dr. Weaver. The virus stock was prepared in 10-day-old embryonated chicken eggs, and the mouse lethal dose 50 (MLD_50_) was determined in our previous studies (20). A peptide array spanning the HA protein of the influenza virus A/New York/18/2009 (H1N1)pdm09 was obtained from BEI Resources (NR-19245). This peptide library comprised 139 peptides, each containing 14 or 15 amino acids and overlapping with adjacent peptides by 11 amino acids.

### Plasmid construction

To generate the plasmid containing the enhanced green fluorescent protein (eGFP) fused in-frame with the nanoluciferase (Nluc) gene (pCI-eGFP-Nluc), the eGFP gene was amplified by PCR from the pEGFP-C1 plasmid (Clontech) and cloned in-frame at the 5′ end of the Nluc gene (GenBank: AFI79290.1), which had previously been inserted into the pCI plasmid (Promega, #E1731). A flexible GGGGS linker was inserted between the eGFP and Nluc proteins. The HA gene from the swine-origin H1pdm09 virus A/swine/Iowa/A01202099/2011 (GenBank: AFM46639.1), with a Flag-tag sequence (DYKDDDDK) fused to its carboxy terminus, was codon-optimized for *Sus scrofa* and synthesized commercially. All genes were cloned into the pCI plasmid backbone (Promega, #E1731) between the *NheI* and *NotI* restriction sites. The recombinant plasmid was propagated in *E. coli* DH5α and purified using the GigaPrep Kit (Zymo Research, #D4204).

### Linear DNA amplification

Linear DNA fragments containing the human cytomegalovirus (CMV) major immediate-early enhancer/promoter region, transgene, and SV40 late poly(A) signal were generated using a two-round PCR amplification strategy. In the first round, Primer Set 1 was used, which consisted of a forward primer annealing upstream of the CMV enhancer and a reverse primer annealing downstream of the poly(A) signal (Table 1). Each primer contained an additional 20-nucleotide non-plasmid adaptor sequence to serve as binding sites for the second round of amplification. In the second round, Primer Set 2, complementary to the overhang sequences introduced in the first round, was used to re-amplify the purified PCR products. All primers were designed using OligoAnalyzer 2.0 software. PCRs were performed using DreamTaq PCR Master Mix (Thermo Scientific, #K1081). The thermocycler program consisted of one cycle at 98°C for 30 s, followed by 40 cycles at 98°C for 10 s, 65°C for 20 s, and 72°C for 50 s and a final extension at 72°C for 75 s. The resulting linear DNA products were concentrated by centrifugation using a polyethersulfone centrifugal concentrator (Sartorius, #VS0642) at 3,000 × *g* for 30 min, followed by phenol-chloroform extraction. Final products were eluted in nuclease-free water.

**TABLE 1:** Primers for production of the linear DNA amplicons by PCR^*a*^

Primer name	Sequence (5′ → 3′)
Primer set 1 Rev	AAGGAATCAACGAGACAGGGGTTGTTGTTAACTTGTTTATTGCAGC
Primer set 1 Fwd	AAAGACACAACCACAGAGCGCATGTTGGCATTGATTATTGACTAG
Primer set 2 Rev	AAGGAATCAACGAGACAGGG
Primer set 2 Fwd	AAAGACACAACCACAGAGCG

^*a*^ Adaptor sequences added to primer set 1 are underlined.

### Lipid nanoparticle preparation

The lipid components used for nanoparticle formulation included DLin-MC3-DMA (MC3; Nanosoft Polymer, #25325), 1,2-dioleoyl-3-trimethylammonium-propane (DOTAP; Avanti Polar Lipids, #890890), distearoylphosphatidylcholine (DSPC; Avanti Polar Lipids, #850365), cholesterol (Sigma-Aldrich, #C3045), and 1,2-dimyristoyl-rac-glycero-3-methoxypolyethylene glycol-2000 (DMG-PEG2000; Avanti Polar Lipids, #880151). MC3, DOTAP, DSPC, cholesterol, and DMG-PEG2000 were individually dissolved in absolute ethanol and combined at a molar ratio of 42:10:8:38:2, respectively. The lipid mixture was combined with an acidification buffer of 25 mM sodium acetate buffer (pH 4.0) containing circular plasmid or linear DNA at a volume ratio of 3:1 (aqueous:ethanol) with a total flow rate of 4 ml per minute using a microfluidic mixer (NanoGenerator Flex-M system, PreciGenome) as previously described (20). The resulting LNP-DNA particles were analyzed for physicochemical properties, including particle size, polydispersity index, and encapsulation efficiency as described before (20).

### *In vitro* transfection efficiency evaluation

To evaluate transfection efficiency, LNP-encapsulated linear or circular plasmid DNA encoding the eGFP-NLuc fusion protein was added directly to HEK-293T cells seeded in 24-well plates 24 h prior, with 500 ng of encapsulated DNA used per well. At 36 h post-transfection, eGFP expression was assessed by fluorescence microscopy using the Nikon Eclipse Ts2R system (Nikon Instruments Inc.). To quantify the percentage of eGFP-expressing cells, transfected cells were dislodged by treatment with 0.25% trypsin-EDTA for 5 min, washed in complete DMEM medium, resuspended in 200 μL FACS buffer, and analyzed by flow cytometry (CytoFLEX LX, Beckman Coulter). For luciferase quantification, a separate set of transfected cells was harvested and lysed in 20 μL RIPA buffer for 30 min on ice. Lysates were clarified by centrifugation at 17,000 × *g* for 15 min, diluted 1:1,000 in nuclease-free water, and 50 μL of the diluted sample was mixed with luciferase substrate (Promega, #N1110). Luminescence was measured using a luminescent plate reader (Synergy LX, BioTek). To further assess the expression of the eGFP-NLuc fusion protein, remaining cell lysates were resolved by SDS-PAGE and transferred to polyvinylidene fluoride (PVDF) membranes. Immunoblotting was performed using an anti-GFP antibody (Santa Cruz Biotechnology, #sc-9996), followed by goat anti-mouse IgG HRP conjugation (Invitrogen, #31430). Detection was performed using the Clarity Western ECL Substrate (Bio-Rad, #10026384) for 5 min, and protein bands were visualized using the ChemiDoc MP Imaging System (Bio-Rad). β-actin was used as a loading control.

### Evaluation of immune responses and protection against H1N1 CA09 virus in mice

Forty-five six-week-old female BALB/c mice were obtained from Charles River Laboratories and randomly assigned to three groups (*n* = 15 per group). Groups 1 and 2 received two doses of 10 µg LNP-encapsulated plasmid DNA (LNP-P) or LNP-encapsulated linear DNA (LNP-L), respectively, on days 0 and 21. Both vaccine formulations contained the HA protein of the swine-origin H1pdm09 virus A/swine/Iowa/A01202099/2011 and were administered intramuscularly into the left and right thigh muscles. Group 3 received no treatment and served as the non-vaccinated (NV) control. Whole blood was collected weekly from all mice, except on day 7, to assess antibody responses. On day 50 post-vaccination, five mice per group were euthanized, and spleens were harvested and cryopreserved for T-cell response analysis. The remaining 10 mice per group were challenged intranasally with 20 MLD_50_ of the CA09 virus. Five mice per group were monitored daily for 14 days to evaluate weight loss and survival. Mice that lost equal to or greater than 25% of their initial body weight (measured at the time of challenge) were humanely euthanized and recorded as deceased. The remaining five mice per group were euthanized on day five post-challenge, and lungs were collected. One half of each lung was stored at −80°C for subsequent quantification of viral loads. The other half was fixed in 10% neutral-buffered formalin for 24 h, paraffin-embedded, and sectioned at a thickness of 4 μm. Lung sections were stained with hematoxylin and eosin (H&E) for histological evaluation, following previously described parameters (20). A separate set of slides was used for RNA *in situ* hybridization (ISH) with a probe specific to viral nucleoprotein transcripts (Advanced Cell Diagnostics, #579001) and the RNAscope 2.5 HD Reagent Kit-BROWN (Advanced Cell Diagnostics, #322300), as previously described (33). To quantify the ISH-positive cells, tissue sections were scanned using an Aperio ScanScope CS slide scanner (Leica Biosystems), and whole-slide images were analyzed using QuPath v0.7.0. Regions of interest were manually annotated, and ISH-positive cells were quantified using the Positive Cell Detection algorithm. Cells were classified as ISH-positive when the mean 3,3′-diaminobenzidine (DAB) optical density exceeded 0.2. The percentage of ISH-positive cells within each annotated region was reported.

### Hemagglutination inhibition assay

Hemagglutination inhibition (HI) titers of serum samples collected at various time points post-vaccination were measured against the H1N1 CA09 virus as previously described (34). Titers were recorded as the reciprocal of the highest dilution that completely inhibited hemagglutination and are expressed as geometric mean titers with standard error of the mean. Samples that did not show HI activity at the initial dilution (1:10) were assigned an arbitrary titer of 1:5 for graphical and statistical analysis.

### Whole-virus indirect enzyme-linked immunosorbent assay (ELISA)

Nunc Maxisorp enzyme-linked immunosorbent assay (ELISA) plates (Thermo Fisher Scientific, #446469) were coated overnight at 4°C with sucrose-purified CA09 virus diluted in 100 mM carbonate-bicarbonate buffer (pH 9.6) at a concentration of 128 HA units per well. The following day, plates were blocked for 1 h at room temperature with a blocking buffer consisting of phosphate-buffered saline (PBS) supplemented with 25% (vol/vol) ChonBlock buffer (Chondrex Inc., #9068) and 5% (wt/vol) non-fat milk. Serum samples were diluted 1:100 in blocking buffer, and 100 µL of each diluted sample was added to duplicate wells of the coated plate. The plates were incubated for 1 h at room temperature, washed five times with PBS containing 0.1% Tween-20 (PBS-T20), and then incubated with HRP-conjugated secondary antibodies specific to different mouse IgG subclasses, including IgG (Invitrogen, #31430), IgG1 (Southern Biotech, #1071-05), IgG2a (Southern Biotech, #1080-05), and IgG2b (Southern Biotech, #1090-05). All secondary antibodies were diluted 1:2,000 in blocking buffer. After five washes with PBS-T20, TMB substrate (Alpha Diagnostic Intl., #80093-1000) was added and incubated for 5 min at room temperature, followed by the stop solution (Alpha Diagnostic, #80100). Absorbance was measured at 450 nm using a microplate reader (BioTek). Samples with an optical density (OD) that exceeded the detection limit of the plate reader were assigned an arbitrary OD value of 4.5 for graphical and statistical analysis.

### Interferon-gamma enzyme-linked immunospot assay

The interferon-gamma enzyme-linked immunospot (IFN-γ ELISpot) assay was performed as previously described (20). Cryopreserved mouse splenocytes were thawed, washed once with RPMI supplemented with 10% fetal bovine serum (cRPMI), and plated at a density of 2.5 × 10^5^ cells per well in PVDF 96-well plates (MilliporeSigma, #MSIPS4W10) pre-coated with an anti-mouse IFN-γ monoclonal antibody (Mabtech, #3321-3-250). Cells were separately stimulated with four overlapping peptide pools (pool 1, peptides 1–40; pool 2, peptides 36–75; pool 3, peptides 71–110; and pool 4, peptides 106–139). Each peptide was used at a final concentration of 250 ng per well. The following day, IFN-γ spots were detected using a biotinylated anti-IFN-γ detection antibody (Mabtech, #3321-6-250), streptavidin-alkaline phosphatase (SouthernBiotech, #7105-04) and BCIP/NBT substrate (Mabtech, #3650-10). Spots were counted using an automated ELISpot reader (AID iSpot). The spot count from unstimulated wells was subtracted from that of peptide-stimulated wells, and results are presented as IFN-γ-secreting cells per 2.5 × 10^5^ splenocytes.

### Flow cytometry

Cryopreserved splenocytes were thawed, washed, resuspended in cRPMI, and plated in duplicate in round-bottom 96-well plates at a density of 0.5 × 10^6^ live cells per well. Cells were stimulated with a pool consisting of 139 overlapping peptides, with the final concentration of each peptide being 250 ng per well. A separate set of unstimulated cells served as negative controls. After 1 h incubation at 37°C, brefeldin A solution (BioLegend, #420601) was added to each well at a final concentration of 1 μg/mL. Six hours later, cells were collected for flow cytometry analysis. Cells were first stained with Zombie NIR viability dye (BioLegend, #423105), followed by two washes with FACS buffer. Surface staining was performed using antibodies against CD3ε (BioLegend, #100232), CD4α (BioLegend, #100433), and CD8α (BioLegend, #100728), followed by three washes with FACS buffer. Cells were then fixed and permeabilized using Cytofix/Cytoperm (BD Biosciences, #51-2090KZ), followed by three washes with Perm/Wash buffer (BD Biosciences, #51-2091KZ). Intracellular staining was performed using antibodies specific to IFN-γ (BioLegend, #505810), TNF-α (BioLegend, #506345), and IL-2 (BioLegend, #503806). After three final washes with perm/wash buffer, cells were resuspended in 200 μL FACS buffer and analyzed using a CytoFlex cytometer (Beckman Coulter). Approximately 100,000 events were recorded per sample. Data were analyzed using FlowJo software (BD Biosciences).

### Virus titration

Virus titers in lungs collected on day 5 post-challenge with the H1N1 CA09 virus were determined by titration on MDCK cells. Titers were calculated by the Reed and Muench method and expressed as log_10_ TCID_50_ per 100 mg. Samples with undetectable infectious virus were assigned a value of 0 log₁₀ for graphical and statistical analysis.

### Statistics

Graphs and statistical analyses were performed using GraphPad Prism (GraphPad Software Inc., Version 10.6.0). Before statistical analysis, HI titers and viral infectious titers were log_2_ and log_10_ transformed, respectively. The data were analyzed separately for each individual sampling date. One-way analysis of variance was used to evaluate overall differences among the three groups, and Tukey’s *post hoc* test was used for pairwise comparisons. Statistical differences were considered significant at *P* < 0.05.

## RESULTS

### LNP-encapsulated linear DNA produces lower protein expression than LNP-encapsulated plasmid DNA

**Fig 1:**
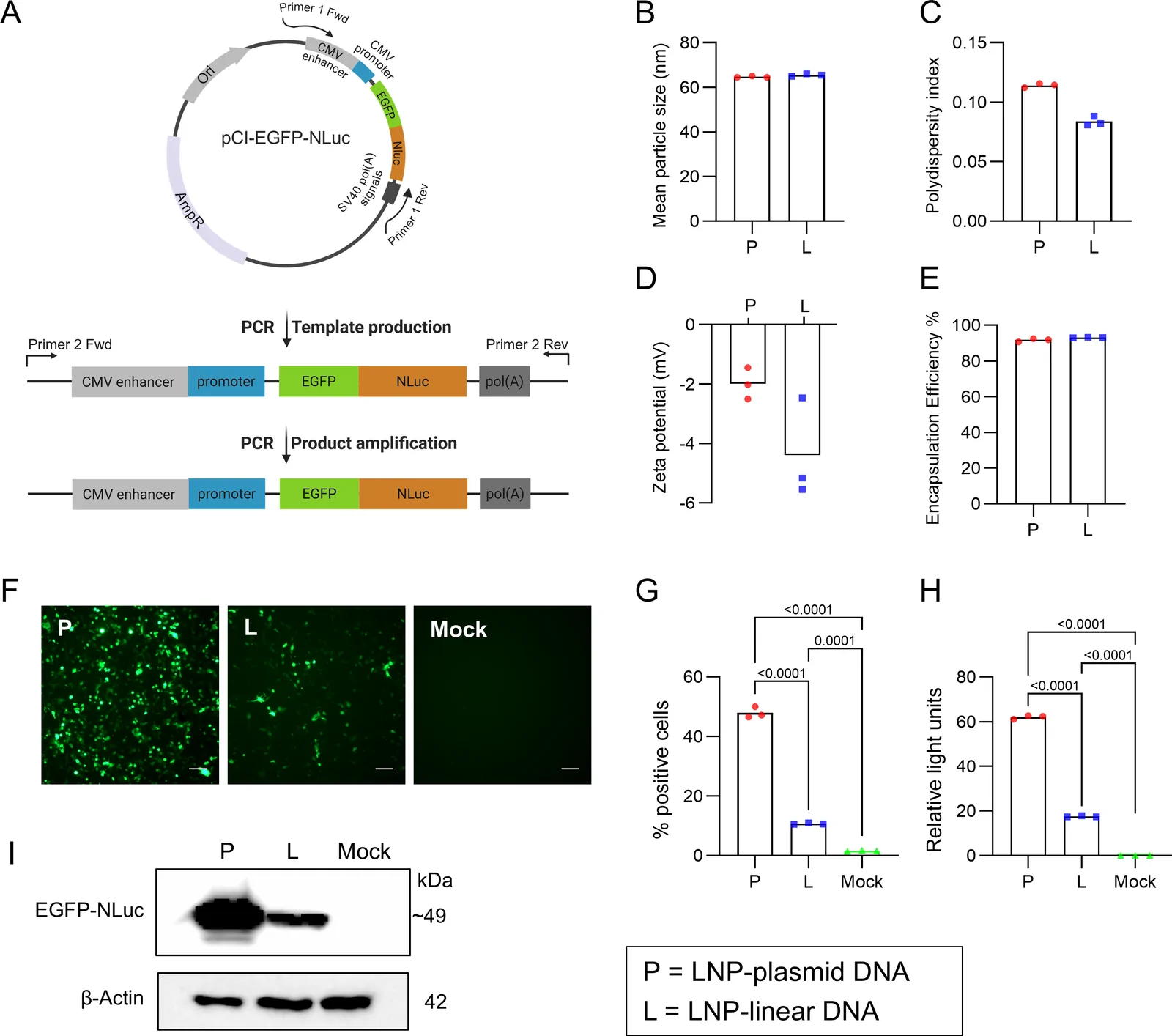
Physicochemical properties and transfection efficiency of the LNP-DNA vaccines. (**A**) Schematic representation of the generation of a linear eGFP-NLuc DNA construct by PCR amplification from the pCI-eGFP-NLuc plasmid. (**B–E**) Physicochemical properties of LNP-plasmid DNA and LNP-linear DNA formulations, including average particle diameter (**B**), polydispersity index (**C**), zeta potential (**D**), and encapsulation efficiency (**E**). (**F–I**) Protein expression *in vitro*. HEK-293T cells seeded in 24-well plates were transfected with 500 ng of encapsulated circular plasmid or linear DNA encoding the eGFP-NLuc fusion protein. At 36 h post-transfection, GFP-expressing cells were observed under a microscope (**F**), and cells were collected and the frequencies of GFP-positive cells were quantified by flow cytometry (**G**). Additionally, luciferase activity was measured by NanoLuc luciferase assay (**H**), and protein expression was confirmed by Western blotting using an antibody specific to GFP (**I**). In panels **B–E and G-H**, symbols represent data from independent replications, while bars represent the means.

To evaluate the feasibility of LNP-mediated delivery of linear DNA, a linear DNA construct was generated by PCR amplification from the pCI-eGFP-NLuc plasmid using a two-step amplification approach (Fig. 1A). The DNA amplicon was approximately 2,400 bp and contained 3 essential elements: the CMV immediate-early enhancer/promoter region, the eGFP-NLuc fusion gene, and the SV40 poly(A) signal. When encapsulated into LNPs, the linear (LNP-L) and plasmid (LNP-P) DNA formed spherical nanoparticles with an average particle size of approximately 65 nm (Fig. 1B) and mean polydispersity index values around 0.1 (Fig. 1C). Both LNP-L and LNP-P exhibited a slightly negative surface charge, with zeta potentials of −4.4 mV for LNP-L and −2.0 mV for LNP-P (Fig. 1D). The encapsulation efficiency (EE%) for LNP-L and LNP-P was 93.1% and 91.7%, respectively (Fig. 1E). Thus, LNPs effectively encapsulated both forms of DNA.

At 36 h post-transfection into HEK-293T cells, GFP-positive cells were clearly visible in cultures that received either LNP-L or LNP-P (Fig. 1F). However, cells transfected with LNP-L exhibited a significantly lower percentage of GFP-positive cells than those treated with LNP-P (10.6% vs 47.9%) (Fig. 1G). Since the transgene includes the NLuc gene, luciferase activity was measured to quantitatively assess protein expression. The bioluminescent intensity of cells transfected with LNP-L was three times lower than those transfected with LNP-P (Fig. 1H). Protein expression was further confirmed by Western blot analysis. A protein band corresponding to the expected size of the eGFP-NLuc fusion protein was detected in cells transfected with either LNP-P or LNP-L. However, the band intensity was visibly weaker in cells transfected with LNP-L (Fig. 1I). These findings demonstrate that while LNPs can efficiently deliver both plasmid and linear DNA, LNP-L produced significantly lower transgene expression than LNP-P.

### LNP-linear DNA induces systemic antibody responses in mice

Next, we utilized the mouse model for IAV to assess the immunogenicity and protective efficacy of the LNP-L and LNP-P vaccine formulations. A linear DNA amplicon and circular DNA plasmid encoding the HA protein of the H1N1pdm09 virus were constructed and encapsulated into LNPs. The resulting formulations, LNP-L and LNP-P, were transfected into HEK-293T cells to assess antigen expression. Indirect immunofluorescence analysis showed lower HA expression in cells transfected with LNP-L than in those transfected with LNP-P (Fig. S1), consistent with the results obtained using the eGFP-NLuc reporter antigen.

**Fig 2:**
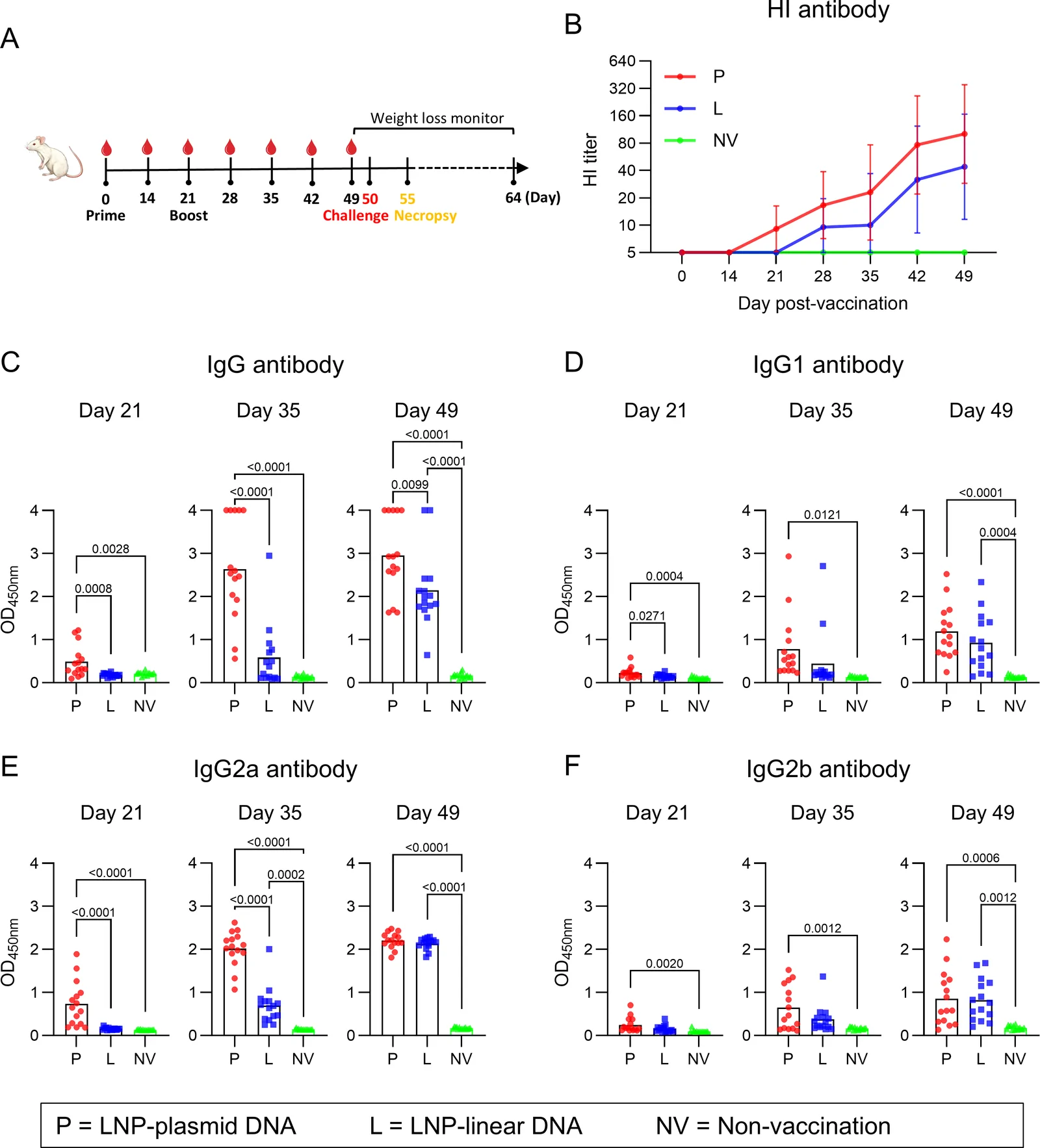
Humoral immune responses after immunization. (**A**) Female BALB/c mice were randomly assigned to three experimental groups (*n* = 15 per group) and immunized intramuscularly on days 0 and 21 with 10 µg of either LNP-plasmid DNA or LNP-linear DNA vaccine. Blood samples were collected weekly. On day 50, mice were challenged intranasally with 20 MLD_50_ of the CA09 virus and monitored daily for body weight for 14 days post-challenge. (**B**) Hemagglutination inhibition (HI) antibody titers against the CA09 virus. Samples with undetectable HI antibodies at the initial dilution of 1:10 were assigned a titer of 1:5 for graphical presentation. Data are shown as geometric mean titers ± standard deviation. (**C–F**) CA09-specific antibody subtypes, including IgG (**C**), IgG1 (**D**), IgG2a (**E**), and IgG2b (**F**), were measured using CA09 whole-virus-based ELISAs. Results are presented as OD450 values. In panels **C–F**, symbols represent data from individual mice, while bars represent group means.

To assess immune responses, two groups of mice were immunized with either LNP-P or LNP-L, while a third group served as a non-vaccinated (NV) control (Fig. 2A). Serum HI antibodies were measured against the H1N1pdm09 CA09 strain, which shares 97.7% HA amino acid similarity with the vaccine immunogen. Mice immunized with LNP-P had detectable HI titers by day 21, prior to the booster immunization, and the titers rapidly increased after the booster, reaching a mean of 1:80 on day 49 (Fig. 2B). In contrast, HI titers were only detected in the LNP-L group starting on day 28, 1 week after the booster dose. From day 28 onward, the mean HI titers in the LNP-L group were approximately one log_2_ lower than those of the LNP-P group, but the differences were not statistically significant (Fig. 2B). HI titers were not detected in the NV group at any time point.

CA09-specific IgG was measured by ELISA to further characterize the humoral immune response. In the LNP-P group, serum samples exhibited significantly higher OD values than those from the NV group at all three sampling days, with OD values significantly increased in samples collected on subsequent days (Fig. 2C). In the LNP-L group, only samples collected on day 49 showed significantly higher OD values than those in the NV group. On each sampling day, OD values of samples from the LNP-P group were consistently higher than those from the LNP-L group.

ELISA results for CA09-specific IgG subclasses, including IgG1, IgG2a, and IgG2b, followed the same trend observed for total virus-specific IgG (Fig. 2D through F). In the LNP-P group, the mean OD values for these three IgG subclasses were significantly greater than those in the NV group at all three time points tested. In the LNP-L group, mean OD values for IgG1 and IgG2b were only significantly greater than those of the NV group for samples collected on day 49 (Fig. 2D and F), while mean OD values for IgG2a were significantly greater than the NV group on both days 35 and 49 (Fig. 2E). Notably, for all three IgG subclasses, the mean OD values on day 49 were not significantly different between the LNP-P and LNP-L groups.

### LNP-linear DNA induces T-cell responses in mice

**Fig 3:**
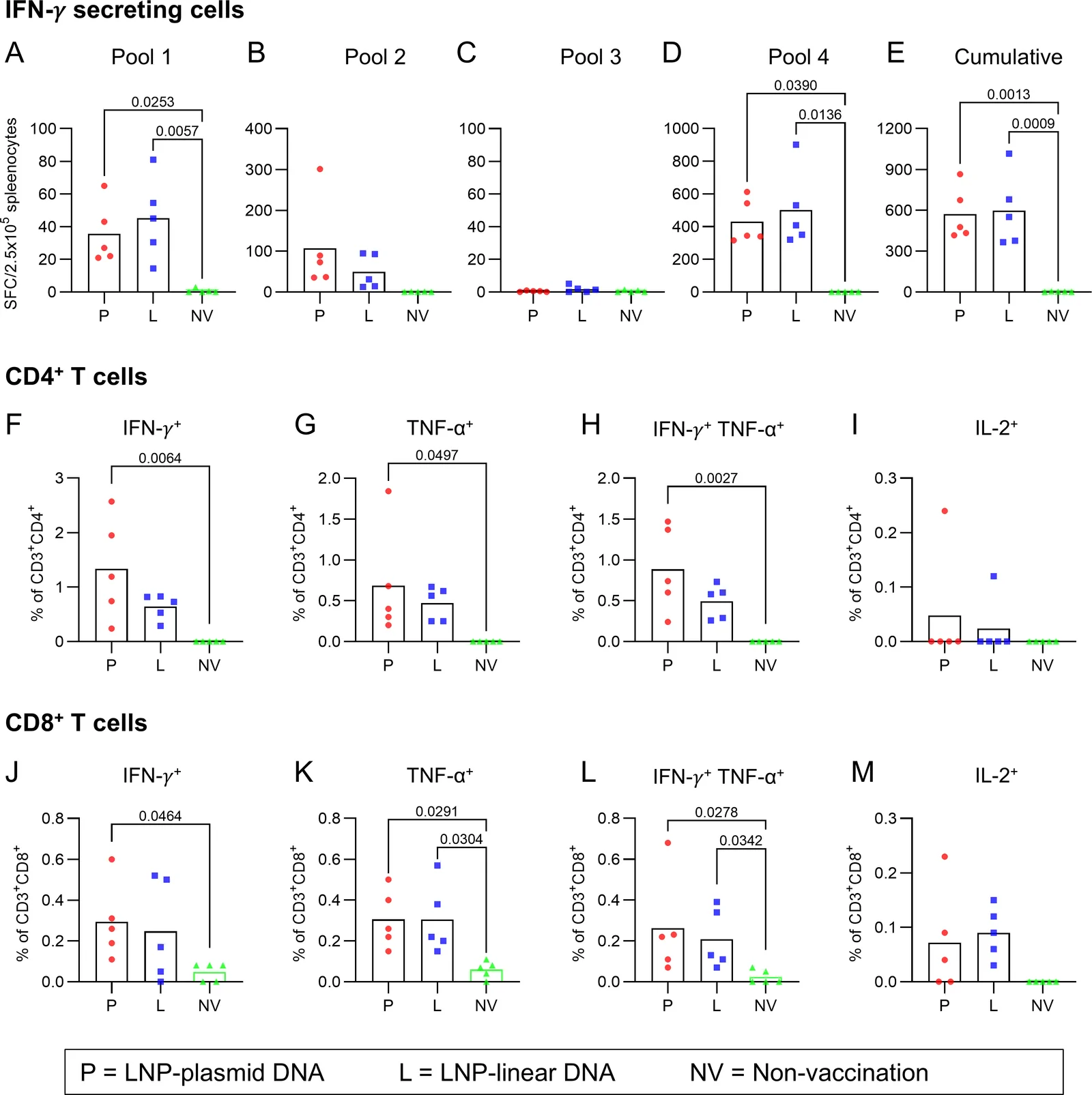
Cell-mediated immune responses after immunization. (**A–E**) IFN-γ ELISpot assay. Splenocytes collected on day 49 post-vaccination were stimulated with four overlapping synthetic peptide pools spanning the HA protein of the genetically similar H1N1 A/New York/18/2009 virus. The number of IFN-γ-secreting cells was quantified by IFN-γ ELISpot and expressed as spot-forming cells (SFC) per 2.5 × 10⁵ cells after background subtraction. (**F–M**) Intracellular cytokine staining. Splenocytes were stimulated for 6 h with an overlapping peptide pool encompassing the entire HA protein of H1N1 A/New York/18/2009. Cells were stained for surface markers, including CD3, CD4, and CD8, followed by intracellular cytokine staining for IFN-γ^+^, TNF-α^+^, and IL-2^+^. Cells were analyzed by flow cytometry and gated on CD3^+^CD4^+^ and CD3^+^CD8^+^ populations expressing individual or dual cytokines. In all panels, symbols represent data from individual mice, and bars represent group means.

To evaluate cellular immune responses, splenocytes from five mice per group were collected on day 49 (1 day before challenge) and stimulated with four pools of overlapping peptides spanning the HA antigen, and numbers of antigen-specific IFN-γ-secreting cells (IFN-γ-SC) were quantified by the IFN-γ ELISpot assay. IFN-γ-SC were virtually undetectable in the NV group, regardless of the peptide pool used for stimulation. In contrast, splenocytes from the LNP-P and LNP-L groups produced significant numbers of IFN-γ-SC when stimulated with peptide pools 1 and 4, with no statistically significant difference between them (Fig. 3A and D). Notable numbers of IFN-γ-SC were also observed in both LNP-P and LNP-L groups following stimulation with peptide pool 2; however, these differences were not statistically significant compared to the NV group (Fig. 3B). IFN-γ-SC were barely detectable when splenocytes were stimulated with peptide pool 3, which spans amino acids 71 to 105 of the HA protein (Fig. 3C). To assess the overall T-cell response against the HA antigen, cumulative IFN-γ-SC counts across all four peptide pools were calculated. Both LNP-P and LNP-L groups exhibited significantly higher cumulative IFN-γ-SC counts than the NV group, with no significant difference between them (Fig. 3E).

Next, flow cytometry was used to further characterize the T-cell response. Splenocytes collected on day 49 were stimulated with a single peptide pool spanning the entire HA protein and stained for 3 surface markers (CD3, CD4, and CD8) and 3 intracellular cytokines (IFN-γ, TNF-α, and IL-2). The LNP-P group showed significantly higher frequencies of CD4^+^ T cells expressing IFN-γ, TNF-α, and dual IFN-γ/TNF-α than the NV group (Fig. 3F through H). In the LNP-L group, CD4^+^ T cells expressing IFN-γ, TNF-α, and dual IFN-γ/TNF-α were also detected, but the differences were not statistically significant compared to the NV group (Fig. 3F through H). Similarly, splenocytes from the LNP-P group showed significantly higher frequencies of CD8^+^ T cells expressing IFN-γ, TNF-α, and dual IFN-γ/TNF-α than the NV group (Fig. 3J). On the other hand, the LNP-L group had significantly higher frequencies of TNF-α^+^ and dual IFN-γ^+^/TNF-α^+^ CD8^+^ T cells compared with NV (Fig. 3K and L). The frequencies of CD4^+^ nor CD8^+^ T cells expressing IL-2 were not statistically different between the immunized mice and the NV control (Fig. 3I and M).

### LNP-linear DNA provides complete protection against lethal challenge in mice

**Fig 4:**
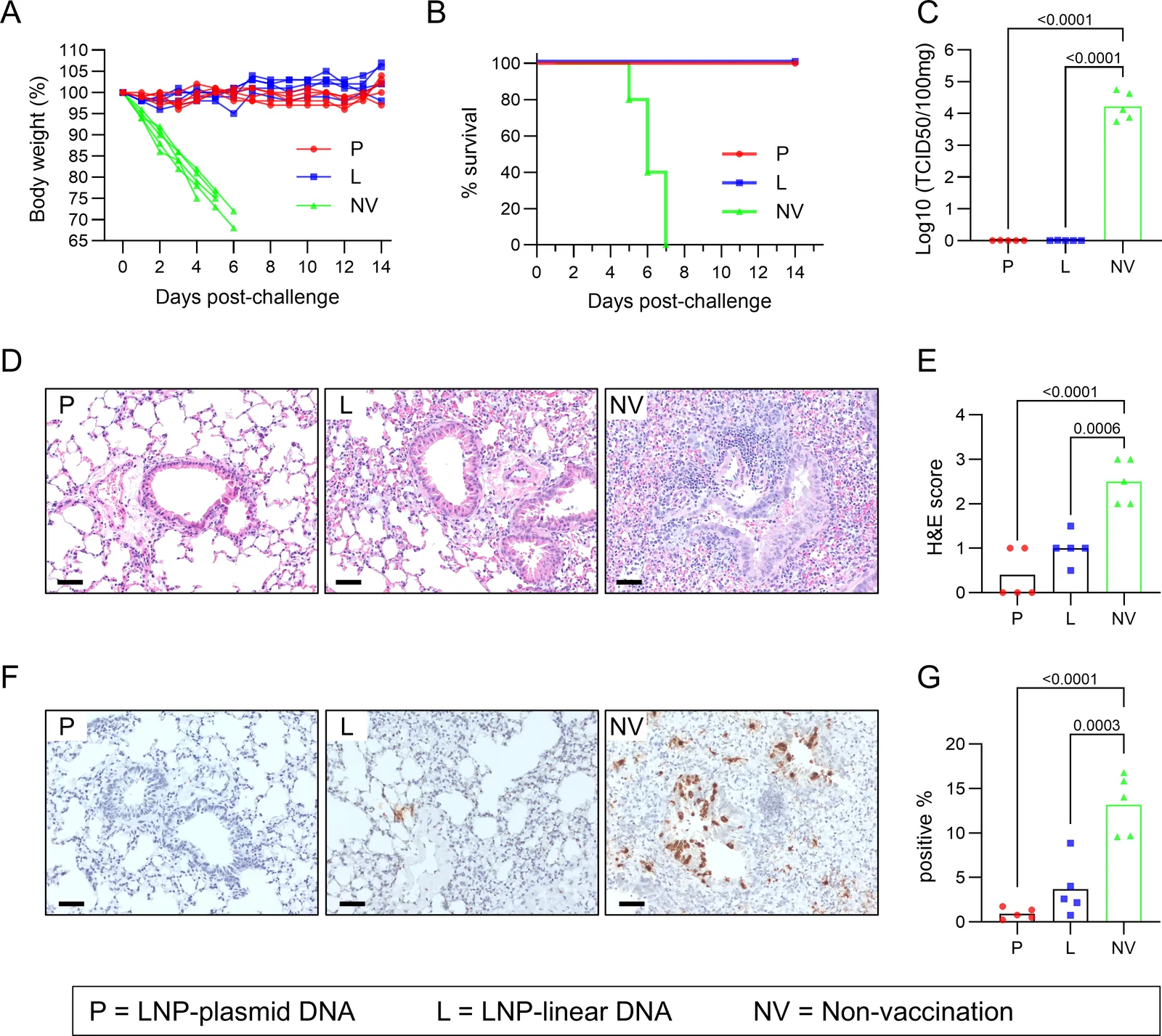
Protection following challenge infection. On day 50 post-vaccination, mice were challenged via intranasal inoculation with 20 MLD_50_ of the H1N1pdm09 A/California/07/2009 virus. Five animals from each group were monitored daily for body weight loss (**A**) and survival (**B**) for 14 days. An additional five animals from each group were euthanized on day 5 post-challenge. One-half of the lung was collected, and viral loads were quantified by titration in MDCK cells (**C**). The remaining lung tissue was fixed in formalin and processed for histopathology (H&E) and *in situ* hybridization (ISH). Representative images of histopathological changes in the lung are shown in panel **D**, with composite microscopic lesion scores in panel **E**. Representative images of lung sections stained by ISH are shown in panel **F**, with corresponding ISH scores in panel **G**. Scale bar = 50 µm. In panel **A**, each line represents an individual animal. In panels **C, E, and G**, symbols represent data from individual mice while bars represent group means.

On day 50, mice were intranasally challenged with 20 MLD_50_ of the mouse-adapted H1N1pdm09 CA09 strain. Following the challenge, mice in the NV group began to lose weight starting on day 2 (Fig. 4A). By day 7 post-challenge, all mice in the NV group had reached humane endpoints and were euthanized (Fig. 4A and B). In contrast, mice vaccinated with either LNP-P or LNP-L did not lose weight and survived throughout the 14-day observation period (Fig. 4A and B).

Five mice per group were euthanized on day 5 post-challenge to evaluate lung viral loads and pathology. The lungs of mice in the NV group had high viral titers ranging from 10^3.75^ to 10^4.75^ TCID_50_ per 100 mg of tissue. In contrast, infectious virus was not detected in the lungs of LNP-P or LNP-L immunized mice (Fig. 4C). Regarding lung pathology, mice from the NV group showed widespread inflammatory infiltration, epithelial damage, and alveolar consolidation. In contrast, lung sections from the LNP-P and LNP-L groups displayed preserved tissue structure, minimal inflammation, and intact bronchiolar epithelium (Fig. 4D). As a result, both immunized groups had significantly lower histopathology scores compared to the NV group (Fig. 4E).

*In situ* hybridization (ISH) was used to detect viral NP transcripts in lung sections. In the NV group, NP-positive cells were abundantly detected in the parenchyma and bronchiolar epithelium (Fig. 4F). Conversely, lung sections from the LNP-P group showed no detectable NP-positive cells. In the LNP-L group, NP-positive cells were sporadically detected in the lung sections. As a result, the NV group had significantly higher ISH scores than both the LNP-P and LNP-L groups (Fig. 4G).

## DISCUSSION

Bacterial plasmids are widely used as vectors for DNA vaccine development. However, conventional bacterial plasmids often contain an antibiotic resistance gene, which poses safety concerns (35). DNA amplicons produced by PCR offer a safer alternative approach. In this study, we used the mouse model for IAV to evaluate a DNA vaccine containing a linear DNA amplicon encapsulated in LNP. Mice are readily available, easy to handle, and supported by a wide range of immunological reagents. Immunization with the HA antigen of IAV alone is sufficient to induce complete protection (32). Moreover, HI antibody titers serve as a reliable correlate of vaccine-induced immunity (36). Together, these features make the mouse model of IAV a valuable tool for preclinical vaccine research. Using this system, we demonstrated that mice receiving a prime-boost immunization with the open-ended linear DNA expression cassette encapsulated in LNP were fully protected against a lethal challenge with the H1N1pdm09 strain.

The linear DNA amplicon used in this study contained only three essential elements: the CMV major immediate-early enhancer/promoter region, transgene, and SV40 late poly(A) signal. This simplified design allowed for efficient production of the expression cassette using standard PCR methods. When encapsulated in LNPs, the resulting formulation, LNP-L, exhibited physicochemical properties, such as particle size, polydispersity index, zeta potential, and encapsulation efficiency, comparable to those of the circular plasmid (LNP-P).

One major difference between LNP-P and LNP-L is that LNP-L expressed significantly lower amounts of antigen when transfected into cell cultures *in vitro*. Lower transgene expression from linear DNA compared to circular plasmid DNA has been reported in multiple studies (37, 38), presumably because it is rapidly degraded by intracellular nucleases (39, 40). While plasmid DNA maintains higher and more sustained expression for up to 7 days, protein expression from linear-DNA transfection typically peaks at approximately 48 h and then declines (8). The expression of linear DNA cassettes can be improved by modifying the 5′ and 3′ termini. For instance, incorporation of DNA targeting sequences into linear DNA cassettes, designed to facilitate nuclear translocation, enhances transgene expression (41). In this study, our primary objective was to assess the feasibility of delivering a linear DNA expression cassette using LNPs. As we could detect significant transgene expression in cells receiving the LNP-L, we did not explore the possibility of modifying the linear DNA termini to improve transgene expression.

In this study, mice immunized with the LNP-L vaccine developed antibody responses later than those immunized with the LNP-P vaccine. This observation is consistent with previous studies reporting weaker antibody responses in animals immunized with open-ended linear DNA compared with plasmid DNA, regardless of whether the linear DNA was delivered intramuscularly with electroporation or intradermally by gene gun (42, 43). The delayed humoral immune responses observed in the LNP-L group may be due to lower levels of antigen expression. It has been reported that reduced antigen expression likely limits antigen availability for germinal center formation and B-cell expansion, thereby delaying the development of humoral immunity (44). As a result, antigen-specific antibody responses in mice immunized with the LNP-L vaccine were not detectable until after booster immunization.

However, the magnitude of cellular immune responses did not differ significantly between the LNP-L and LNP-P groups. One possible explanation is that splenocytes were collected on day 49 post-vaccination, when the LNP-L group had already developed significant HI titers (1:40). It is therefore possible that if cell-mediated immunity had been assessed earlier, particularly before booster immunization, the LNP-L group might have exhibited weaker cellular immune responses than the LNP-P group.

Although the onset of the humoral response was delayed in mice immunized with the LNP-L, by day 49, the magnitudes of virus-specific serum antibody subclasses, including IgG1, IgG2a, and IgG2b, were comparable to those observed in mice immunized with the LNP-P vaccine. Because cytokines produced by CD4^+^ T cells regulate immunoglobulin class switching, antibody subclass profiles are often used as surrogate indicators of the cytokine milieu and the balance of Th1/Th2-type help (45). In mice, switching to IgG1 is influenced by Th2 cytokines such as IL-4 and IL-5, while switching to IgG2a and IgG2b is influenced by Th1 cytokines, including IFN-γ (46, 47). Therefore, the simultaneous detection of IgG1, IgG2a, and IgG2b in the serum of mice receiving LNP-L and LNP-P suggests these vaccines induced both Th1 and Th2 responses. This interpretation is further supported by intracellular cytokine staining of splenocytes collected on day 49 post-vaccination, which showed that the frequencies of CD4^+^ T cells producing IFN-γ, TNF-α, or both cytokines (IFN-γ^+^/TNF-α^+^) were not statistically different in mice immunized with either LNP-L or LNP-P vaccines. We also detected appreciable frequencies of antigen-specific CD8^+^ T cells producing IFN-γ and TNF-α in both immunized groups, indicating that both vaccine formats elicited CD8^+^ effector responses. Although cytokine production does not directly measure cytolytic activity, IFN-γ/TNF-α–producing CD8^+^ T cells are commonly used as an indicator of functional cellular immunity following vaccination. Together with the IFN-γ ELISpot data, these findings support the conclusion that, after two immunizations, LNP-L induces T-cell responses of similar magnitude and functional profile to those elicited by LNP-P in mice.

In summary, this study demonstrates that LNPs can effectively encapsulate a linear DNA expression cassette and that immunization of mice with the LNP-encapsulated linear DNA amplicon induces protective immunity. Unlike conventional plasmid-based DNA vaccines, the linear DNA cassette can be rapidly manufactured without bacterial fermentation, eliminating the need for antibiotic selection markers and reducing the risk of contamination with bacterial components such as endotoxins. However, the expression of transgenes delivered by the linear DNA amplicon is significantly lower than that delivered by a circular plasmid, leading to delayed immune responses. Therefore, further research is warranted to enhance transgene expression and improve the immunogenicity of linear DNA-based vaccines.

## Data Availability

All data directly supporting the findings of this study are presented within the article and its supplemental material. The sequences of the H1N1pdm09 virus used in this study are available from the National Center for Biotechnology Information, accession number KF715130.1.

## SUPPLEMENTAL MATERIAL

Figure S1 msphere.00135-26-s0001.tif *In vitro* expression of HA antigen.

ASM does not own the copyrights to Supplemental Material that may be linked to, or accessed through, an article. The authors have granted ASM a non-exclusive, world-wide license to publish the Supplemental Material files. Please contact the corresponding author directly for reuse.
